# A Behavior Change Wheel-Based Nursing Intervention to Improve Maternal Management and Child Outcomes Among Preschool Children with Attention-Deficit/Hyperactivity Disorder

**DOI:** 10.3390/children13070935

**Published:** 2026-07-16

**Authors:** Alaa Mujallad, Fatma Ahmed Elsobky, Hala Mohammed Yasin, Rimas M. Alharbi, Reema H. Mohammed, Najwa A. Alamri, Shahad A. Alsomali, Eman A. Alsoulami, Marwa A. Shahin

**Affiliations:** 1 Department of Nursing, College of Applied Medical Sciences, University of Jeddah, Jeddah 21589, Saudi Arabia; afmojallad@uj.edu.sa (A.M.); hmyassin@uj.edu.sa (H.M.Y.);; 2 Department of Pediatric Nursing, Faculty of Nursing, Benha University, Benha 13511, Egypt; 3 Nursing Program, Batterjee Medical College, Jeddah 21442, Saudi Arabia; 4 Maternal and Newborn Health Nursing Department, Faculty of Nursing, Menoufia University, Shibin Al Kawm 32511, Egypt

**Keywords:** attention-deficit/hyperactivity disorder, behavior change wheel, nursing intervention, maternal management, child outcomes, preschool children

## Abstract

**Background:** Attention-Deficit/Hyperactivity Disorder (ADHD) is one of the most prevalent neurodevelopmental disorders among children and is associated with significant behavioral, academic, and social challenges. Caregiver-focused behavioral interventions have gained increasing attention as effective non-pharmacological approaches for improving child outcomes and enhancing parental management skills. Thus, this study aimed to evaluate the potential effectiveness of behavior change wheel (BCW)-based nursing intervention in improving maternal behavioral management practices, as well as child outcomes involving ADHD symptoms among preschool children aged 3–6 years with attention-deficit/hyperactivity disorder. **Methods:** A one-group pretest–posttest quasi-experimental design was conducted among 55 mothers and their children diagnosed with ADHD at Thawat Center and King Abdullah Center for Disability Services in Jeddah, Saudi Arabia. The study did not include a control group. Participants were recruited using a convenience sampling technique. Data were collected using a sociodemographic questionnaire, ADHD Rating Scale IV—Preschool Version, the BCW Intervention Compliance Questionnaire, and the Parental Knowledge and Attitude Questionnaire. The intervention was implemented over eight weeks and included educational sessions, behavioral skills training, motivational enhancement, role-play activities, and follow-up reinforcement. **Results:** The preliminary findings from the one-group pre/posttest quasi-experimental study revealed statistically significant improvements in children’s inattentive and hyperactivity symptoms following the intervention (*p* < 0.001). Mothers also demonstrated significant improvements in perceived knowledge, attitudes, and compliance with BCW components after program implementation (*p* < 0.001). The proportion of mothers with satisfactory perceived knowledge increased from 21.8% pre-intervention to 94.5% post-intervention, while positive attitudes increased from 23.6% to 98.2%. In addition, compliance with BCW components improved from 30.9% before the intervention to 94.5% after implementation. **Conclusions:** The BCW-based nursing intervention was associated with pre–post improvement in maternal caregiving outcomes and reduced ADHD symptoms among preschool children. These preliminary findings from the one-group pretest–posttest quasi-experimental study suggest the potential value of theory-informed, caregiver-focused nursing interventions in pediatric and community healthcare settings. However, due to the one-group pretest–posttest design without a control group, causal inferences cannot be made. Further controlled studies are needed to confirm these associations and establish causal inferences.

## 1. Introduction

Attention-Deficit/Hyperactivity Disorder (ADHD) is one of the most prevalent neurodevelopmental disorders in childhood and is characterized by persistent patterns of inattention, hyperactivity, and impulsivity that negatively affect children’s functioning across multiple domains. According to the Diagnostic and Statistical Manual of Mental Disorders, Fifth Edition (DSM-5), ADHD symptoms are developmentally inappropriate, occur in more than one context, and result in significant impairment in social, academic, and daily activities. Children with ADHD commonly experience difficulties in sustaining attention, controlling impulses, regulating activity levels, and developing appropriate social relationships, making ADHD one of the most frequent causes of referral to psychological and psychiatric services during childhood [1]. Because many symptoms emerge during the preschool years, early recognition and parent-focused behavioral management are considered fundamental for improving developmental outcomes and reducing the long-term impact of ADHD.

Although ADHD is commonly diagnosed after children enter formal education, the preschool years provide a critical opportunity for early identification and intervention. ADHD affects approximately 2–5% of preschool children, and longitudinal studies indicate that many children who exhibit ADHD symptoms during this period continue to experience clinically significant symptoms throughout school age and later developmental stages. These findings highlight the importance of early identification and timely intervention to improve long-term developmental outcomes. In Saudi Arabia, ADHD has a reported prevalence of approximately 8% among children and adults, emphasizing the need for effective early management approaches within healthcare services [2,3,4].

Early childhood is particularly suitable for intervention because the developing brain demonstrates greater neuroplasticity compared with later developmental periods. During the preschool years, rapid brain growth and functional development enhance children’s ability to adapt to environmental influences and structured interventions. This increased capacity for neural modification provides an important opportunity for behavioral approaches to strengthen self-regulation, adaptive behaviors, and social functioning among children with ADHD. Consequently, early interventions targeting behavioral difficulties during this period may have long-term benefits for children’s developmental outcomes [5,6,7].

The management of ADHD requires accurate assessment because diagnosis depends on clinical evaluation rather than a single diagnostic test. Identification of ADHD involves collecting information from multiple sources, including parents, teachers, and healthcare professionals, together with developmental history and behavioral assessment tools. Moreover, careful differential diagnosis is essential because some medical conditions may present with symptoms resembling ADHD. Recent evidence suggests that children with bronchial asthma may exhibit increased attention difficulties compared with healthy children, emphasizing the importance of comprehensive clinical assessment to distinguish ADHD from attention problems secondary to chronic medical conditions [1,8].

Preschool children spend most of their time within the family environment, and parents represent the primary influence on children’s daily behaviors and emotional regulation. Mothers, particularly, play a central role in implementing behavioral management strategies, establishing routines, reinforcing appropriate behaviors, and responding to challenging behaviors. However, caring for a child with ADHD may increase maternal stress, emotional burden, and difficulties in maintaining consistent parenting practices. Previous evidence indicates that mothers of children with ADHD may experience more negative parenting practices, lower parenting confidence, and psychological challenges compared with mothers of children without ADHD. Therefore, improving maternal skills and supporting caregivers are essential components of effective early ADHD management [9,10].

Current ADHD management commonly involves a combination of pharmacological and non-pharmacological approaches. Although pharmacological treatment effectively reduces core ADHD symptoms, its use in preschool children may be associated with concerns related to adverse effects, variable treatment response, adherence difficulties, and uncertainty regarding long-term functional outcomes. As a result, behavioral approaches have gained increasing importance, and international guidelines recommend behavioral parental training (BPT) as a first-line intervention for preschool children with ADHD. Parent-focused behavioral interventions aim to improve caregivers’ understanding of ADHD, strengthen parenting skills, establish consistent routines, and enhance positive reinforcement strategies [2,11,12].

Despite the effectiveness of behavioral parental interventions, their implementation in real-life settings depends greatly on caregivers’ ability to apply recommended strategies consistently. Applying behavioral techniques in daily routines may remain challenging for some caregivers, particularly when they face stress and competing demands. Nursing-led psychoeducational and behavioral interventions have demonstrated positive effects on caregiver knowledge, parenting practices, stress reduction, and child behavioral outcomes. However, these interventions may benefit from a structured theoretical framework that guides intervention development and supports sustained parental engagement in behavioral management. The behavior change wheel (BCW) provides such a framework by offering a systematic approach to identify appropriate intervention strategies and promote behavioral change among caregivers [13,14,15,16].

The behavior change wheel (BCW), developed by Michie and colleagues, is centered on the capability–opportunity–motivation–behavior (COM-B) model, which proposes that behavioral change occurs through the interaction of capability, opportunity, and motivation. Capability refers to the psychological and physical ability to perform a behavior, opportunity includes environmental and social conditions that support behavior, and motivation involves the cognitive and emotional processes that influence actions. This framework provides a systematic approach for designing interventions by linking behavioral determinants with appropriate intervention strategies [17,18,19].

In the present study, the BCW framework guided the development of a nursing intervention targeting mothers of preschool children with ADHD. The intervention was designed to strengthen maternal behavioral management by applying BCW-based strategies, including education, training, modeling, persuasion, and environmental restructuring. Education and training aimed to enhance mothers’ capability by improving their knowledge of ADHD and developing practical parenting skills. Modeling and persuasion were used to strengthen mothers’ confidence and motivation to apply positive behavioral management strategies consistently. Environmental restructuring supported mothers’ opportunity to implement these strategies by promoting structured home routines and a supportive environment. Through these components, the intervention aimed to improve maternal behavioral management practices and consequently enhance behavioral outcomes among preschool children with ADHD.

Despite a large and growing body of literature, existing reviews frequently examine pharmacological and non-pharmacological interventions separately or focus mainly on treatment efficacy outcomes without sufficient attention to clinical application and implementation processes. This indicates a need for approaches that integrate evidence-based interventions with structured frameworks to support effective implementation and sustained caregiver involvement [3].

The behavior change wheel (BCW) has been widely applied in health behavior interventions; however, its use in nursing interventions targeting mothers of preschool children with ADHD has received limited attention. In Saudi Arabia, published studies have primarily focused on parental knowledge, awareness, perceptions, and educational support rather than theory-guided behavioral interventions. For example, ref. [20] examined the impact of an educational intervention on mothers’ awareness and communication with their children with ADHD, whereas ref. [21] assessed parental knowledge and awareness of ADHD symptoms among Saudi children. To our knowledge, no published study in Saudi Arabia has evaluated a BCW-based nursing intervention designed to improve maternal behavioral management among mothers of preschool children with ADHD. This study was therefore undertaken to address this gap by evaluating a theory-guided nursing intervention and its effects on maternal behavioral management and child behavioral outcomes.

Improving maternal behavioral management during the preschool years may help to reduce ADHD-related behavioral difficulties, strengthen parent–child interactions, and promote positive developmental outcomes. Accordingly, the present study aimed to develop and evaluate a behavior change wheel-based nursing intervention for mothers of preschool children with Attention-Deficit/Hyperactivity Disorder.

Aim of the study: To investigate the potential effect of a behavior change wheel-based nursing intervention on maternal management, as well as child outcomes involving ADHD symptoms among preschool children with attention-deficit/hyperactivity disorder.

Study Hypothesis:

Children whose mothers receive the behavior change wheel-based nursing intervention will show a significant reduction in ADHD symptoms compared with the baseline prior to the intervention.

Mothers who receive the behavior change wheel-based nursing intervention will demonstrate significantly improved perceived knowledge, attitude, and management practices for childhood ADHD post-intervention compared with the baseline prior to the intervention.

## 2. Materials and Methods

### 2.1. Study Design

A one-group pretest–posttest quasi-experimental design was employed to evaluate the potential effectiveness of the behavior change wheel-based nursing intervention in improving maternal management practices and reducing ADHD symptoms among preschool children.

### 2.2. Study Setting

The study was conducted at Thawat Center and King Abdullah Center for Disability Services in Jeddah, Saudi Arabia. Both centers provide educational and developmental support services for children with behavioral and developmental disorders.

### 2.3. Sample and Recruitment

A convenience sampling technique was used to recruit 55 mothers and their children diagnosed with ADHD. The inclusion criteria of the studied sample include preschool children aged 3–6 years with a diagnosis of ADHD. ADHD diagnosis was confirmed based on the documented medical records of the children, with the diagnosis previously established by qualified psychiatrists according to standard clinical diagnostic criteria. Mothers were willing to participate in the intervention sessions and able to attend scheduled assessments and training sessions. Medication status was not used as an eligibility criterion or controlled as part of the intervention.

The exclusion criteria of the study sample include children with severe psychiatric or medical comorbidities and mothers unable to complete the intervention program. Eligible participants were consecutively enrolled from both centers during the study period based on the predefined inclusion criteria. No a priori allocation of participants to either center was established. Rather, recruitment reflected the number of eligible mothers attending each center and their willingness to participate. Therefore, the number of participants enrolled from each center differed according to the availability of eligible cases during the data collection period. Recruitment continued until the required total sample size was attained. In total, 15 study participants were recruited from Thawat Center, and 40 study participants were recruited from King Abdullah Center for Disability Services in Jeddah, Saudi Arabia.

#### Sample Size Calculation

Based on previous literature [2], considering a significance level of 5% and a study power of 80%, the required sample size was calculated using the following formula:

*n* = ((Zα/2 + Zβ)^2^ × 2(SD)^2^)/(d^2^)

where SD represents the standard deviation of the outcome measure obtained from a previous study, Zα/2 = 1.96 for a 5% significance level, Zβ = 0.84 for 80% power, and d represents the expected mean difference based on previous evidence. Using SD = 4.02 and an expected difference (d) = 2.15,

*n* = ((1.96 + 0.84)^2^ × 2(4.02)^2^)/(2.15^2^) = 54.8

Therefore, the minimum required sample size was approximately 55 participants.

### 2.4. Data Collection Tools

#### 2.4.1. Sociodemographic Questionnaire

A structured questionnaire was utilized to gather demographic information about both the child and the mother. It included items such as the child’s age, gender, and birth order, as well as the mother or guardian’s age, educational level, and occupation. It also assessed whether the family had a history of ADHD or other mental health disorders. The instrument was developed following a review of relevant literature, including El-Nagger (2017) [22].

#### 2.4.2. ADHD Rating Scale IV—Preschool Version

The ADHD Rating Scale-IV Preschool Version assesses ADHD symptoms using a modified form of the ADHD Rating Scale-IV originally developed by DuPaul et al. (1998) and adapted for preschool children by McGoey et al. (2007) [23]. The instrument consists of 18 items corresponding to the DSM-IV-TR diagnostic criteria for ADHD (American Psychiatric Association, 2000) [24]. The 18 items contain those for inattention (9 items) and hyperactivity/impulsivity (9 items).

The original scale employs a four-point Likert response format: 0 = Never, 1 = Sometimes, 2 = Often, 3 = Very Often. However, prior to data collection, and to facilitate mothers’ understanding and enhance response accuracy, the researchers adopted a modified three-point response format in which the categories Often and Very Often were merged into a single category. Accordingly, responses were scored as 1 = Never, 2 = Sometimes, and 3 = Often. All data collection, scoring procedures, and statistical analyses were conducted using this modified three-point format.

The inattention and hyperactivity/impulsivity domain scores range from 9 to 27. Symptom severity is classified as mild (9–14), moderate (15–20), or high (21–27). The overall ADHD symptom score ranges from 18 to 54 and is categorized as mild (18–29), moderate (30–41), or high (42–54).

The content validity of the adapted instrument was established through evaluation by a panel of five experts in pediatric nursing, child psychiatry, and psychology. The experts assessed the relevance, clarity, comprehensiveness, and cultural appropriateness of each item. Necessary modifications were made based on their feedback. The Item-Level Content Validity Index (I-CVI) was calculated as the proportion of experts rating each item as either 3 or 4 on a 4-point relevance scale. The average I-CVI across all items was 0.956, indicating excellent content validity.

Construct validity was assessed by evaluating the suitability of the data for factor analysis using the Kaiser–Meyer–Olkin (KMO) measure of sampling adequacy and Bartlett’s test of sphericity. The KMO value was 0.941, exceeding the recommended threshold of 0.60, indicating excellent sampling adequacy. Bartlett’s test of sphericity was statistically significant (χ^2^ = 891.43, *p* < 0.05), demonstrating that the correlation matrix was appropriate for factor analysis. These findings supported the construct validity of the adapted instrument.

Regarding reliability, previous psychometric evaluations conducted by McGoey et al. (2007) [23] demonstrated satisfactory reliability for the original ADHD Rating Scale-IV Preschool Version, with reliability coefficients ranging from 0.80 to 0.95. Since the response format was modified in the present study, internal consistency reliability was reassessed using Cronbach’s alpha coefficient in the current sample. The modified instrument demonstrated excellent internal consistency, yielding a Cronbach’s alpha coefficient of 0.904 for the overall scale, indicating that the adapted version maintained satisfactory reliability among the study participants.

#### 2.4.3. BCW Intervention Compliance Questionnaire

The BCW Intervention Compliance Questionnaire was developed by the researchers based on the behavior change wheel (BCW) framework and the capability–opportunity–motivation–behavior (COM-B) model proposed by Michie et al. (2011) [17], in addition to evidence derived from behavioral management literature and caregiver-focused intervention studies for preschool children with ADHD [2,12,25,26]. The instrument was designed to assess maternal compliance with the behavioral components targeted by the intervention, specifically capability, opportunity, and motivation.

Item generation was undertaken through a comprehensive review of the BCW and COM-B theoretical frameworks with empirical studies addressing parental training, behavioral management strategies, and caregiver interventions for preschool children with ADHD [2,12,17,25,26]. Based on this review, an initial pool of items was developed to ensure adequate representation of the three COM-B domains and to capture mothers’ adherence to the behavioral management practices promoted during the intervention. Each item was systematically mapped to its corresponding COM-B construct to ensure conceptual alignment with the behavioral determinants specified within the BCW framework.

The preliminary version of the questionnaire comprised 34 items distributed across the three COM-B domains. Subsequently, the instrument underwent expert panel review to evaluate item relevance, clarity, and comprehensiveness. Following expert review and pilot testing, four items were removed due to redundancy and ambiguity, resulting in a final 30-item questionnaire. The final instrument consisted of three subscales: capability (10 items), opportunity (10 items), and motivation (10 items). All items were formulated to reflect mothers’ adherence to behavioral management practices relevant to the targeted COM-B domains and intervention objectives.

Content validity was evaluated by a panel of five experts in pediatric nursing, behavioral psychology, psychiatric nursing, and community health nursing. The experts assessed the questionnaire in terms of relevance, clarity, simplicity, and consistency with the BCW framework. The Item-Level Content Validity Index (I-CVI) was calculated as the proportion of experts rating each item as either 3 or 4 on a 4-point relevance scale in relation to the intended COM-B domains. The average I-CVI across all items was 0.960, indicating excellent content validity. Based on the experts’ recommendations, minor linguistic modifications were made to improve clarity and cultural appropriateness.

Construct validity was assessed by evaluating the suitability of the data for factor analysis using the Kaiser–Meyer–Olkin (KMO) measure of sampling adequacy and Bartlett’s test of sphericity. The KMO value was 0.781, exceeding the recommended threshold of 0.60, indicating adequate sampling adequacy. Bartlett’s test of sphericity was statistically significant (χ^2^ = 237.02, *p* < 0.05), demonstrating that the correlation matrix was appropriate for factor analysis. In addition, the items were theoretically mapped to the COM-B model constructs (capability, opportunity, and motivation) to ensure conceptual consistency and adequate representation of the targeted behavioral determinants. These findings supported the construct validity of the adapted instrument.

Regarding reliability, the internal consistency of the BCW Intervention Compliance Questionnaire was assessed using Cronbach’s alpha coefficient. The total scale demonstrated good reliability, with an alpha value of 0.873, indicating satisfactory internal consistency among the items.

In terms of scoring, each item was rated dichotomously, where 1 indicated a correct or compliant response and 0 indicated an incorrect or non-compliant response. The total score ranged from 0 to 30, with higher scores indicating greater compliance with BCW-based behavioral management practices. Domain scores ranged from 0 to 10 and were categorized as low (0–4; <50% of the maximum domain score), moderate (5–7; 50–75% of the maximum domain score), or high (8–10; >75% of the maximum domain score), based on percentage-based classification approaches commonly used in compliance research [27]. For the overall score interpretation, total scores of 15–30 (≥50% of the maximum attainable score) indicated satisfactory or adequate compliance, whereas scores of 0–14 (<50% of the maximum attainable score) indicated unsatisfactory or inadequate compliance. These cut-off points were determined based on the proportion of the maximum attainable score.

#### 2.4.4. Parental Knowledge and Attitude Questionnaire

The Parental Knowledge and Attitude Questionnaire was used to assess mothers’ perceived knowledge and attitudes regarding the management of Attention-Deficit/Hyperactivity Disorder (ADHD) among preschool children. The questionnaire was developed by the researchers based on the recommendations and evidence presented by Daley et al. (2018) [28] concerning behavioral interventions and parental involvement in ADHD management. The instrument consisted of 12 items distributed equally across two domains: perceived knowledge (6 items) and attitude (6 items).

The perceived knowledge domain was designed to evaluate mothers’ self-reported understanding of essential ADHD-related concepts rather than objective factual knowledge. Given that the questionnaire employed a Likert-type agreement scale, this domain primarily assessed mothers’ perceived understanding and awareness of ADHD and its management. The six items addressed several key areas such as recognition of ADHD symptoms, differentiation between inattention and hyperactivity, awareness of behavioral management strategies, understanding of environmental influences on children’s behavior, knowledge of the role of positive reinforcement, and awareness of the neurodevelopmental nature of ADHD. Sample items included: I understand the main symptoms of ADHD in young children, I know the difference between inattention and hyperactivity in ADHD, and I understand that ADHD is a neurodevelopmental disorder rather than a consequence of poor parenting. Higher scores in this domain reflected greater perceived knowledge and understanding related to ADHD management.

The attitude domain assessed mothers’ beliefs, confidence, willingness, and motivation toward implementing ADHD management strategies. Specifically, the items explored mothers’ confidence in managing ADHD symptoms, beliefs regarding the effectiveness of behavioral interventions, willingness to consistently apply recommended strategies, perceptions of the importance of parental involvement, attitudes toward seeking professional support, and motivation to acquire new skills to support their children’s development. Sample items included: I feel confident in my ability to manage my child’s ADHD symptoms, I believe that behavioral interventions can effectively improve my child’s functioning, and I am willing to consistently implement the behavioral strategies learned during the intervention sessions. Higher scores indicated more positive attitudes toward ADHD management.

Responses to all questionnaire items were rated using a four-point Likert scale ranging from 0 to 3, where 0 = strongly disagree, 1 = disagree, 2 = agree, and 3 = strongly agree. Scores for each domain ranged from 0 to 18. Higher scores indicated greater perceived knowledge and more positive attitudes regarding ADHD management.

For interpretative purposes, each domain score was categorized into two levels. In the perceived knowledge domain, scores ≥ 9 were considered indicative of satisfactory perceived knowledge, whereas scores < 9 reflected unsatisfactory perceived knowledge. Similarly, in the attitude domain, scores ≥ 9 indicated a positive attitude toward ADHD management, while scores < 9 reflected a negative attitude.

The content validity of the instrument was established through evaluation by a panel of five experts in pediatric nursing, child psychiatry, and psychology. The experts assessed each item for relevance, clarity, comprehensiveness, and cultural appropriateness. Necessary modifications were made based on their recommendations. The Item-Level Content Validity Index (I-CVI) was calculated as the proportion of experts who rated each item as either 3 or 4 on a 4-point relevance scale. The average I-CVI was 0.967 for both the perceived knowledge and attitude domains, indicating excellent content validity.

Construct validity was assessed by evaluating the suitability of the data for factor analysis using the Kaiser–Meyer–Olkin (KMO) measure of sampling adequacy and Bartlett’s test of sphericity. The KMO values were 0.687 for the perceived knowledge domain and 0.762 for the attitude domain, exceeding the recommended threshold of 0.60 and indicating adequate sampling adequacy. Bartlett’s test of sphericity was statistically significant for both domains (χ^2^ = 385.65, *p* < 0.05 for perceived knowledge; χ^2^ = 278.66, *p* < 0.05 for attitude), demonstrating that the correlation matrices were appropriate for factor analysis. These findings supported the construct validity of the instrument. Additionally, construct validity was supported by organizing the questionnaire into two theoretically distinct domains of perceived knowledge and attitude which were consistent with the conceptual framework underlying ADHD management and the objectives of the present study.

The reliability of the instrument was assessed by evaluating internal consistency using Cronbach’s alpha coefficient. The Cronbach’s alpha values were 0.880 for the perceived knowledge domain and 0.900 for the attitude domain, indicating good-to-excellent internal consistency reliability.

### 2.5. Pilot Study

A pilot study was conducted prior to the main data collection on 10% of the study sample (*n* = 6 mothers) who met the study inclusion criteria. The pilot participants were recruited from the same study setting and had characteristics similar to those of the target population. The pilot study was undertaken to assess the clarity and comprehensibility of the instrument items, identify any ambiguities, and evaluate the feasibility of the study procedures. Based on the pilot preliminary findings from the one-group study, minor modifications were made to improve the clarity and applicability of the study instruments. Because these participants had been exposed to the study instruments before the main data collection, they were excluded from the final study sample.

### 2.6. Ethical Considerations

Ethical approval was obtained from the Institutional Review Board of the University of Jeddah. Written informed consent was obtained from all participating mothers. Confidentiality, anonymity, and voluntary participation were maintained throughout the study.

### 2.7. Field Organization and Intervention Implementation

To ensure the systematic implementation of the study procedures, participants were recruited from two study settings: Thawat Center and King Abdullah Center for Disability Services. Eligible participants were consecutively enrolled from both centers throughout the study period according to the predefined inclusion criteria. No predetermined quota was assigned to either center. Instead, participant recruitment was based on the availability of eligible mothers attending each center and their willingness to participate in the study. Consequently, the number of participants recruited from each center varied according to the availability of eligible cases during the data collection period. Recruitment continued across both centers until the required total sample size was achieved.

**Figure 1:**
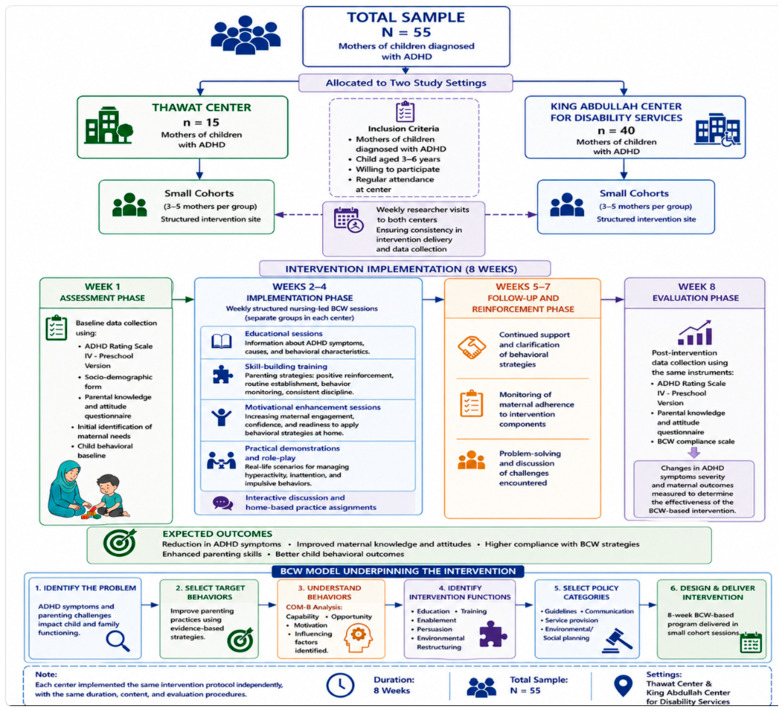
Framework diagram of the study.

To ensure consistency in intervention delivery across the two study centers, all researchers involved in implementing the intervention received standardized training before data collection by the principal researcher and adhered to a unified intervention protocol. The same educational materials, session content, sequence, duration, and teaching strategies were used across both centers. Each center functioned as a structured intervention site, where mothers of children diagnosed with Attention-Deficit/Hyperactivity Disorder (ADHD) were organized into small cohorts to facilitate the delivery of the behavior change wheel (BCW)-based nursing intervention by assigned researchers. The overall framework of the study was presented in Figure 1.

The intervention was implemented over an eight-week period and was organized into the following sequential phases:

Week 1: Baseline Assessment Phase

During the first week, baseline data were collected from all participants prior to the intervention. The assessment included the ADHD Rating Scale IV—Preschool Version, a sociodemographic data sheet, the BCW Compliance Questionnaire and the Parental Knowledge and Attitude Questionnaire. In addition, initial maternal needs were identified, and baseline levels of child behavioral symptoms were established to guide subsequent intervention planning.

Weeks 2–4: Implementation Phase: Behavior Change Wheel-Based Nursing Intervention Guided by the COM-B Model

During this phase, a structured nursing intervention grounded in the behavior change wheel (BCW) framework and the capability–opportunity–motivation–behavior (COM-B) model was implemented to improve maternal caregiving practices and subsequently enhance developmental and behavioral outcomes among preschool children diagnosed with Attention-Deficit/Hyperactivity Disorder (ADHD). Mothers, as primary caregivers, were targeted to strengthen their knowledge, caregiving skills, environmental support, and motivation to sustain positive behavioral management at home.

The intervention was delivered by trained nursing researchers experienced in pediatric health through four weekly sessions. Each session lasted approximately 60–90 min and was conducted in small groups of 3–5 mothers to facilitate interaction, engagement, and individualized support. A combination of educational, behavioral, motivational, and skills-based approaches was employed, with each session linked to relevant COM-B components and BCW intervention functions to ensure theoretical consistency.

A researcher-developed educational booklet was distributed to all participants at the beginning of the intervention. The educational booklet served as the primary instructional resource throughout the intervention. It contained educational information, behavioral observation sheets, reward charts, daily routine templates, and home practice exercises that were actively used during all intervention sessions and home assignments. Examples and worksheets included in the booklet were actively used during group discussions, practical exercises, and home assignments throughout the intervention period.

Each session followed a standardized format consisting of (1) a review of previous home assignments (10–15 min), (2) presentation of new content (20–30 min), (3) practical activities and group discussion (20–30 min), and (4) assignment of home practice tasks (10–15 min).

Attendance was recorded using standardized attendance logs. Mothers who missed a session received an individual review of the missed content before attending subsequent sessions to maintain intervention continuity. To ensure intervention fidelity, all sessions were delivered according to a standardized intervention manual. Identical educational materials, teaching methods, session sequence, and duration were used across both study settings. Researchers completed session checklists after each session to verify implementation of all planned activities, and periodic supervision by the principal researcher ensured adherence to the intervention protocol.

The four intervention sessions were implemented as follows:

Session 1: Psychoeducation

The first session aimed to improve mothers’ understanding of ADHD. Information was presented regarding the nature of ADHD, common symptoms, causes, misconceptions, and the impact of ADHD on children’s daily functioning.

Practical examples of children’s behaviors were presented through case scenarios and audiovisual material. Mothers were asked to identify ADHD symptoms in the presented examples and discuss similar situations experienced with their own children. Mothers completed a short exercise using examples presented in the booklet to identify problematic behaviors exhibited by their own children. Home assignment: Mothers were instructed to observe their children for one week and record ADHD-related behaviors using the behavioral observation sheet provided in the booklet.

Session 2: Parenting Skills and Behavioral Management Training

The second session focused on teaching mothers effective behavioral management strategies. Researchers demonstrated the use of positive reinforcement, verbal praise, and behavior-shaping techniques.

Mothers practiced these skills through scenario-based exercises and role-playing activities. Examples included responding to non-compliance, managing hyperactivity and tantrums, improving attention during learning activities, and establishing daily routines. Participants also practiced completing behavior-monitoring charts and designing individualized reward systems suitable for their children.

Home assignment: Mothers implemented one reward strategy and one structured daily routine at home and documented their experiences using the monitoring forms included in the booklet.

Session 3: Motivation and Emotional Support

The third session addressed maternal stress and emotional challenges associated with caring for children with ADHD. Group discussions allowed mothers to share experiences, difficulties, and successful strategies.

Reflective exercises were conducted to help mothers identify personal strengths and barriers to implementing behavioral strategies. Relaxation exercises, breathing techniques, and self-care practices were demonstrated and practiced during the session. Individual feedback was provided by researchers to enhance mothers’ confidence and encourage continued implementation of learned strategies.

Home assignment: Mothers were asked to continue applying behavioral strategies and record challenges encountered during implementation.

Session 4: Practical Demonstration and Skill Rehearsal

The final session focused on integrating and reinforcing previously learned skills. Researchers first demonstrated appropriate responses to common ADHD-related situations, including impulsivity, hyperactivity, emotional outbursts, and refusal to follow instructions.

Mothers subsequently participated in supervised role-play activities simulating real-life situations encountered at home. Each mother practiced behavioral management techniques while receiving immediate feedback and corrective guidance from researchers. Group problem-solving activities were also conducted to discuss barriers encountered during home implementation and identify feasible solutions.

At the end of the session, mothers developed individualized action plans describing how they would continue implementing behavioral management strategies at home.

Weeks 5–7: Follow-up and Reinforcement Phase

This phase focused on reinforcing acquired skills and supporting continued application of behavioral strategies at home. Mothers discussed their experiences, challenges, and progress in implementing the recommended strategies. Researchers provided clarification, problem-solving support, and feedback to strengthen adherence to intervention components.

Week 8: Post-Intervention Evaluation Phase

At the end of the intervention period, post-intervention data were collected using the same instruments administered at baseline, including the ADHD Rating Scale IV—Preschool Version, the Parental Knowledge and Attitude Questionnaire, and the BCW Compliance Questionnaire. Changes in child behavioral symptoms and maternal outcomes were assessed to determine the effectiveness of the intervention.

### 2.8. Data Analysis

All statistical analyses were performed using SPSS for Windows version 20.0 (SPSS Inc., Chicago, IL, USA). Continuous variables were tested for normality and are presented as the mean ± standard deviation (SD), whereas categorical variables are presented as frequencies and percentages. Pre- and post-intervention comparisons of continuous outcome measures (ADHD total score, mothers’ total perceived knowledge, total attitude, and total compliance scores) were performed using paired *t*-tests. Comparisons of paired binary categorical variables were performed using McNemar’s test, while Pearson’s Chi-square test (or Fisher’s exact test when appropriate) was used to compare categorical variables with more than two categories. Pearson’s correlation coefficient was used to examine the relationships between ADHD symptoms and mothers’ perceived knowledge, attitude, and compliance following the intervention. The magnitude of the intervention effect was estimated using Cohen’s d, calculated as the difference between the pre- and post-intervention means divided by the pooled standard deviation, and 95% confidence intervals were reported. Internal consistency reliability was assessed using Cronbach’s alpha. Statistical significance was set at *p* < 0.05.

## 3. Results

Table 1 demonstrates the reliability and validity assessment of the study instruments. The internal consistency reliability was acceptable for all instruments, with Cronbach’s alpha coefficients ranging from 0.873 to 0.904, indicating good-to-excellent reliability. The content validity results showed high agreement among experts, with I-CVR values ranging from 0.956 to 0. 967. The construct validity was assessed using the Kaiser–Meyer–Olkin (KMO) measure and Bartlett’s test of sphericity. The KMO values ranged from 0.687 to 0.941, indicating adequate-to-excellent sampling adequacy. Bartlett’s test of sphericity was significant for all instruments, supporting the suitability of the data for factor analysis.

Table 2 shows that the highest percentage of children were aged 3–4 years (52.7%), followed by children aged 5–6 years (47.3%). In addition, males and females represented 45.5% and 49.1% of the sample, respectively. Regarding birth order, the highest proportion was of middle children (32.7%), while the lowest was of only children (12.7%). For the mothers’ age, most mothers were aged 30–40 years (43.6%) and the least were over 40 years (21.8%). Concerning education, most mothers had primary education (52.7%), whereas only 7.3% had a bachelor’s degree. Finally, regarding employment status, unemployment represented 43.6%, which was the highest category in this section.

Table 3 demonstrates a comparison of ADHD symptom severity before and after the BCW-based nursing intervention among the studied children, as reported by their mothers. In the inattention domain, 87.3% of the children were classified as having high symptom severity before the intervention, whereas 81.8% were classified as having mild symptom severity after the intervention. Similarly, in the hyperactivity/impulsivity domain, 74.5% of the children had high symptom severity before the intervention, whereas 96.4% had mild symptom severity after the intervention.

Regarding overall ADHD symptom severity, 90.9% of the children were classified as having high symptom severity and 9.1% as having moderate symptom severity before the intervention. Following the intervention, 94.5% were classified as having mild symptom severity, while only 5.5% remained in the moderate severity category (*p* < 0.001).

The mean ADHD Rating Scale score was significantly lower after the intervention (24.9 ± 2.7) than before the intervention (44.2 ± 2.3) (*p* < 0.001). A large pre–post effect size (Cohen’s d = 7.703) was observed, with a 95% confidence interval of 18.353, 20.247.

Table 4 shows the overall perceived knowledge level. Before the intervention, 78.2% of mothers had unsatisfactory perceived knowledge and 21.8% had satisfactory perceived knowledge. After the intervention, 94.5% of mothers achieved satisfactory perceived knowledge, while only 5.5% remained at an unsatisfactory level (*p* < 0.001). The mean knowledge score significantly increased from 6.7 ± 2.5 before the intervention to 13.5 ± 2.5 after the intervention (*p* < 0.001). A large pre–post effect size (2.717) was observed with a 95% confidence interval of −7.747, −5.853.

Table 5 demonstrates the overall attitude. Before the intervention, 76.4% of mothers had a negative attitude and 23.6% had a positive attitude. Following the intervention, 98.2% of mothers demonstrated a positive attitude, while only 1.8% retained a negative attitude (*p* < 0.001). The mean attitude score significantly increased from 7.1 ± 1.8 before the intervention to 13.3 ± 2.5 after the intervention (*p* < 0.001). The effect size was large (2.276) with a 95% confidence interval of −10.611, −7.589.

Table 6 displays mothers’ compliance pre- and post-implementation of the BCW-based nursing intervention (*p*< 0.001). The proportion of mothers with high capability increased from 12.7% before the intervention to 56.4% after the intervention. Similarly, the percentage of mothers with high opportunity increased markedly from 14.5% before the intervention to 80.0% after the intervention, while the proportion of mothers with high motivation rose from 23.6% before the intervention to 70.9% after the intervention. Regarding the overall compliance level, adequate compliance increased from 30.9% before the intervention to 94.5% after the intervention (*p* < 0.001). The mean compliance score increased significantly from 12.6 ± 3.9 before the intervention to 21.7 ± 4.1 after the intervention (*p* < 0.001). The effect size was large (2.821) with a 95% confidence interval of −7.031, −5.369.

Table 7 shows the correlation between ADHD symptoms and mothers’ perceived knowledge, total attitude, and total compliance after implementation of the BCW-based nursing intervention. A statistically significant negative correlation was found between ADHD symptoms and mothers’ perceived knowledge (r = −0.399, *p* = 0.003). Similarly, ADHD symptoms were negatively correlated with mothers’ total attitude (r = −0.434, *p* < 0.001) and total compliance (r = −0.328, *p* = 0.015) A statistically significant positive correlation was observed between mothers’ perceived knowledge and attitude (r = 0.491, *p* < 0.001), as well as between perceived knowledge and compliance (r = 0.451, *p* < 0.001). Mothers’ attitude also showed a significant positive correlation with compliance (r = 0.306, *p* = 0.023).

## 4. Discussion

The present one-group pretest–posttest quasi-experimental study examined pre–post changes in mothers’ perceived knowledge, attitudes, compliance, and preschool children’s ADHD symptom severity following implementation of a behavior change wheel (BCW)-based nursing intervention. The observed improvements suggest the potential value of incorporating theory-driven behavioral frameworks into nursing interventions targeting caregivers of children with ADHD. Overall, favorable changes in maternal caregiving outcomes and child behavioral outcomes were observed following participation in the BCW-based nursing intervention.

The current preliminary findings demonstrated statistically significant pre–post reductions in inattentive symptoms following the intervention. These observed changes may be associated with the intervention’s emphasis on strengthening mothers’ behavioral management skills through education, training, and structured home-based strategies. Increased maternal consistency in implementing behavioral routines and reinforcement strategies may have contributed to the establishment of a more organized and predictable environment, which, in turn, may have supported children’s attentional regulation and been associated with reductions in inattentive symptoms. These preliminary findings are consistent with previous studies suggesting that parent-focused behavioral interventions may be associated with reductions in inattentive behaviors among children with ADHD [2,29]. Moreover, the inclusion of practical demonstrations and role-play activities may have enhanced mothers’ ability to translate their perceived knowledge into caregiving practices within daily-life situations.

Regarding hyperactivity and impulsivity symptoms, statistically significant pre–post reductions were also observed following the intervention. These changes may reflect the BCW framework’s emphasis on enhancing caregiver capability, opportunity, and motivation as conceptualized within the COM-B model. Although caregiver confidence and behavioral management competencies were not directly assessed, the observed improvement in mothers’ compliance may indicate greater engagement with behavioral management strategies, which may have contributed to more consistent responses to disruptive behaviors and been associated with the observed reductions in hyperactive and impulsive symptoms. These preliminary findings are consistent with previous evidence suggesting that structured parental behavioral interventions were associated with reductions in behavioral dysregulation among children with ADHD [29]. Similarly, Chacko et al. (2024) [30] emphasized the potential of psychosocial interventions to influence developmental outcomes, while Lange et al. (2016) [31] reported that parental management training has been associated with improvements in ADHD symptoms and related behavioral outcomes. In addition, Mansourjozan (2019) [32] reported significant improvements in behavioral and psychological outcomes among children with ADHD following an eight-week horse therapy intervention. Although this intervention differed from the present BCW-based nursing intervention, both studies suggest that structured non-pharmacological approaches may be associated with improvements in behavioral outcomes. Collectively, these findings support the potential value of structured non-pharmacological interventions for improving behavioral outcomes among children with ADHD.

The preliminary findings of the present study demonstrated statistically significant pre–post increases in mothers’ perceived knowledge regarding ADHD and behavioral management following the intervention. The observed improvement may be attributable to the educational content delivered throughout the BCW sessions, which provided structured information regarding ADHD symptoms, behavioral manifestations, and management strategies and may have increased mothers’ confidence in their understanding of these topics. This perceived improvement may support more confident caregiving practices; however, the present study assessed perceived rather than objectively tested knowledge.

This finding is broadly consistent with Alseraty et al. (2024) [20], who reported improvements in mothers’ ADHD knowledge following an educational intervention. Similarly, Alhalawany et al. (2021) [33] reported significant improvements in mothers’ ADHD knowledge following the implementation of educational guidelines, with knowledge scores increasing from predominantly unsatisfactory before the intervention to satisfactory immediately afterward and at follow-up. Although these studies assessed knowledge using different instruments and measurement approaches, they similarly demonstrated improvements in knowledge-related outcomes following educational interventions. Because the present study assessed perceived rather than objectively tested knowledge, the observed improvement should be interpreted as reflecting mothers’ increased confidence in their understanding of ADHD rather than as evidence of objectively verified knowledge acquisition.

Concerning maternal attitudes, statistically significant positive pre–post changes were observed following the intervention. This attitudinal change may reflect the influence of the supportive and motivational elements integrated within the BCW framework. Mothers who perceive behavioral interventions as useful and achievable may be more likely to maintain engagement in caregiving practices and adhere to recommended behavioral strategies. It is also plausible that participation in the intervention may have contributed to reductions in feelings of stigma, helplessness, or self-blame that may accompany caring for children with ADHD; however, these constructs were not directly assessed in the present study. These preliminary findings are consistent with behavioral theories emphasizing the importance of caregiver beliefs and self-efficacy in facilitating sustained behavioral change and intervention adherence (Michie et al., 2011) [17]. Similarly, Abd El Moneam et al. (2018) [34] reported that implementing a psychoeducational program for parents of children with ADHD was associated with positive changes in parental attitudes.

Another notable preliminary finding was the statistically significant pre–post increase in mothers’ compliance with BCW components following the intervention. Improvements were observed across all COM-B domains, including capability, opportunity, and motivation. These preliminary findings suggest that participation in the intervention may have helped to address a range of practical and psychological barriers influencing maternal adherence to behavioral management strategies. Furthermore, the structured follow-up, reinforcement sessions, and problem-solving discussions incorporated into the program may have supported continued maternal engagement and strengthened implementation fidelity. The present preliminary findings suggest that theory-informed behavioral interventions may offer potential advantages when multiple determinants of caregiver behavior are addressed simultaneously (Michie et al., 2011) [17]. Similarly, Alhalawany et al. (2021) [33] and Abd El Moneam et al. (2018) [34] reported improvements in mothers’ skills and practices in managing children with ADHD following educational interventions, suggesting that these improvements may have been related to mothers’ increased motivation to learn positive parenting strategies, enhance involvement, and improve their ability to manage children with ADHD.

Beyond statistical significance, the clinical significance of the observed improvements should also be considered. The preliminary findings of the present study suggest that participation in the BCW-based nursing intervention may have practical implications for enhancing maternal caregiving practices and supporting behavioral regulation among preschool children with ADHD. Improvements in mothers’ perceived knowledge, attitudes, and compliance may facilitate more consistent implementation of behavioral management strategies, better structuring of home routines, and more effective responses to children’s behavioral challenges. Likewise, the observed reduction in ADHD symptom severity may reflect potential improvements in children’s attention regulation, behavioral organization, and daily functioning.

Despite these encouraging findings, the results should be interpreted with caution. Although statistically significant changes and large effect sizes were observed across maternal and child-related outcomes, the one-group pretest–posttest quasi-experimental design without a control group limits causal inference. Consequently, alternative explanations, including maturation, testing effects, regression to the mean, and other time-related influences, cannot be excluded. Furthermore, most study outcomes, including maternal perceived knowledge, attitudes, compliance, and children’s ADHD symptom severity, were assessed primarily using mothers’ self-reported measures. Therefore, the observed improvements may reflect a combination of actual changes in caregiving behaviors and measurement-related influences, such as increased awareness, social desirability bias, or participants’ expectations following exposure to the intervention.

Accordingly, these preliminary findings should be regarded as indicative of the potential clinical value of integrating theory-driven behavioral approaches into nursing interventions for caregivers of children with ADHD rather than as definitive evidence of intervention effectiveness. Overall, participation in the BCW-based nursing intervention was associated with favorable pre–post changes in maternal caregiving outcomes and preschool children’s ADHD symptom severity. However, because the study did not include a control group, these findings should not be interpreted as establishing a causal relationship between the intervention and the observed changes. Future randomized controlled trials with larger sample sizes, longer follow-up periods, objective behavioral assessments, and reports from multiple informants are warranted to determine the effectiveness, clinical relevance, and sustainability of BCW-based nursing interventions.

## 5. Strengths of the Study

The present study contributes to the growing evidence supporting caregiver-focused behavioral interventions for ADHD management during early childhood. One of the major strengths of the study is the application of the behavior change wheel and COM-B framework, which provided a structured and theory-driven approach for designing the intervention. In addition, the intervention incorporated multiple components, including education, behavioral training, motivational enhancement, and follow-up reinforcement, thereby addressing several determinants of caregiver behavior simultaneously.

The study also highlights the important role of nursing interventions in supporting family-centered ADHD management and promoting early behavioral regulation among preschool children.

## 6. Limitations

Several limitations should be considered when interpreting the findings of the present study. First, the use of a one-group quasi-experimental design without a control group limits the ability to establish definitive causal relationships between the intervention and the observed outcomes. In the absence of a control group, the observed changes cannot be attributed exclusively to the intervention, as alternative explanations, including maturation effects, repeated testing effects, regression to the mean, expectancy effects, and response bias, cannot be ruled out. Second, the relatively small sample size and the use of convenience sampling may limit the generalizability of the findings to broader populations. Additionally, the study relied primarily on maternal self-report measures, which may have increased the risk of response and social desirability bias. The relatively short follow-up period also limited the ability to evaluate the long-term sustainability of the intervention. The medication status of the children was not considered, although pharmacological treatment may have influenced behavioral outcomes independently of the intervention. Despite these limitations, the study provides preliminary evidence regarding the potential benefits of behavior change wheel-based nursing interventions in improving maternal behavioral management practices and reducing ADHD symptom severity among preschool children.

## 7. Implications for Nursing Practice

The findings of this study emphasize the importance of integrating theory-driven behavioral interventions into pediatric and community nursing practice. Nurses play a central role in caregiver education, behavioral counseling, and early intervention for children with ADHD. The use of BCW-based interventions may strengthen caregivers’ behavioral management skills, improve adherence to behavioral strategies, and enhance child outcomes. Healthcare institutions should consider implementing structured nursing programs based on behavioral change theories to support families of children with ADHD.

## 8. Conclusions

The preliminary findings of this one-group pretest–posttest quasi-experimental study suggest that participation in the BCW-based nursing intervention may be associated with improvements in mothers’ perceived knowledge, attitudes, and compliance regarding the management of Attention-Deficit/Hyperactivity Disorder (ADHD) among preschool children aged 3–6 years. Statistically significant pre–post reductions in children’s inattentive and hyperactivity/impulsivity symptoms were also observed following the intervention. These preliminary findings suggest the potential value of theory-informed nursing interventions in supporting the behavioral management of preschool children with ADHD. Overall, these preliminary findings suggest that BCW-based nursing interventions may have potential value within pediatric and family-centered nursing practice. However, larger controlled studies with longer follow-up periods are needed to determine whether the observed pre–post changes can be attributed to the intervention and to establish their long-term clinical relevance.

Based on the findings of the present study, the following recommendations are suggested:

- Integrate behavior change wheel (BCW)-based nursing interventions into routine pediatric and community healthcare services to support caregivers of children with ADHD.
- Develop structured educational and behavioral training programs for mothers focusing on parenting skills, positive reinforcement, and behavioral regulation strategies.
- Provide continuous follow-up and reinforcement sessions to maintain caregiver adherence and sustain intervention outcomes.
- Incorporate behavioral change theories, particularly the BCW and COM-B framework, into nursing education and professional training programs.
- Encourage multidisciplinary collaboration among nurses, psychologists, pediatricians, and educators to ensure comprehensive ADHD management.
- Future research prioritizes conducting well-designed randomized controlled trials (RCTs) with larger sample sizes and extended follow-up periods to overcome methodological limitations and provide more robust evidence regarding the long-term effectiveness of BCW-based interventions for children with ADHD and their caregivers.

## Figures and Tables

**Table 1:** Reliability and validity of the study instruments.

	Cronbach’s Alpha	Content Validity (I-CVR)	Construct Validity	
			Kaiser–Meyer–Olkin (KMO) Measure	Bartlett’s Test of Sphericity
ADHD Rating Scale	0.904	0.956	0.941	891.43
Mothers’ Perceived Knowledge	0.880	0.967	0.687	385.65
Mothers Total Attitude	0.900	0.967	0.762	278.66
Mothers Total Compliance	0.873	0.960	0.781	237.02

**Table 2:** Percentage distribution of mothers’ and children’s sociodemographic and historical characteristics in the study group (*n* = 55).

Characteristics	N	%
Age of the Child (Years)		
3–4	29	52.7
5–6	26	47.3
Gender of the Child		
Male	25	45.5
Female	27	49.1
Non-binary	3	5.5
Order of Birth		
Firstborn	16	29.1
Middle child	18	32.7
Lastborn	14	25.5
Only child	7	12.7
Age of Mother/Guardian (Years)		
20–30	19	34.5
30–40	24	43.6
Over 40	12	21.8
Level of Education (Mother/Guardian)		
No formal education	1	1.8
Primary education	29	52.7
Secondary education	21	38.2
Bachelor	4	7.3
Occupation (Mother/Guardian)		
Unemployed	24	43.6
Part-time employment	9	16.4
Full-time employment	13	23.6
Self-employed	7	12.7
Retired	2	3.6
Does the family have a history of ADHD or other mental health disorders?		
Yes	5	9.1
No	34	61.8
Unsure	16	29.1

**Table 3:** Comparison of ADHD symptom severity before and after the BCW-based nursing intervention.

Items	Pre BCW Intervention		Post BCW Intervention		Chi-Square/Fisher’s Test
	N	%	N	%	
The inattention Domain					
Mild symptom severity	0	0.0	45	81.8	X^2^ = 93.529, *p* < 0.001 **
Moderate symptom severity	7	12.7	10	18.2	
High symptom severity	48	87.3	0	0.0	
Hyperactivity/impulsivity Domain					
Mild symptom severity	0	0.0	53	96.4	X^2^ = 103.000, *p* < 0.001 **
Moderate symptom severity	14	25.5	2	3.6	
High symptom severity	41	74.5	0	0.0	
Overall ADHD Rating					
Mild symptom severity	0	0.0	52	94.5	X^2^ = 102.500, *p* < 0.001 **
Moderate symptom severity	5	9.1	3	5.5	
High symptom severity	50	90.9	0	0.0	
Mean ± SD	44.2 ± 2.3		24.9 ± 2.7		T = 36.909, *p* < 0.001 **
Effect Size (Cohen’s d)	7.703				
95% CI	[18.353, 20.247]				

T: Paired T-test. ** indicates statistical significance at the 1% level (*p* < 0.01).

**Table 4:** Percentage distribution of mothers according to total perceived knowledge before and after implementation of the BCW-based nursing intervention.

Items	Pre BCW Intervention		Post BCW Intervention		McNemar Test
	*n*	%	*n*	%	
Unsatisfactory knowledge	43	78.2	3	5.5	X^2^ = 4.267, *p* = 0.039 *
Satisfactory knowledge	12	21.8	52	94.5	
Mean ± SD	6.7 ± 2.5		13.5 ± 2.5		T = 15.495, *p* < 0.001 **
Effect size (Cohen’s d)	2.717				
95% CI	[−7.747, −5.853]				

T: Paired T-test. * indicates statistical significance at the 5% level (*p* < 0.05); ** indicates statistical significance at the 1% level (*p* < 0.01).

**Table 5:** Percentage distribution of mothers according to total attitude before and after implementation of the BCW-based nursing intervention.

Items	Pre BCW Intervention		Post BCW Intervention		McNemar Test
	N	%	N	%	
Negative attitude	42	76.4	1	1.8	X^2^ = 8.643, *p* = 0.003 *
Positive attitude	13	23.6	54	98.2	
Mean ± SD	7.1 ± 1.8		13.3 ± 2.5		T = 15.028, *p* < 0.001 **
Effect size (Cohen’s d)	2.276				
95% CI	[−10.611, −7.589]				

T: Paired T-test. * indicates statistical significance at the 5% level (*p* < 0.05); ** indicates statistical significance at the 1% level (*p* < 0.01).

**Table 6:** Percentage distribution of mothers according to total compliance to BCW component before and after implementation of the BCW-based nursing intervention.

Items	Pre BCW Intervention		Post BCW Intervention		Chi-Square/Fisher’s Test
	N	%	N	%	
Wheel component capabilities for reducing ADHD symptoms and risk factors					
Low capability	29	52.7	11	20.0	X^2^ = 24.383, *p* < 0.001 **
Moderate capability	19	34.5	13	23.6	
High capability	7	12.7	31	56.4	
Wheel component opportunities for reducing ADHD symptoms and risk factors					
Low opportunity	32	58.2	3	5.5	X^2^ = 51.082, *p* < 0.001 **
Moderate opportunity	15	27.3	8	14.5	
High opportunity	8	14.5	44	80.0	
Wheel component motivation for reducing ADHD symptoms and risk factors					
Low motivation	28	50.9	4	7.3	X^2^ = 31.154, *p* < 0.001 **
Moderate motivation	14	25.5	12	21.8	
High motivation	13	23.6	39	70.9	
Overall compliance					
Inadequate compliance	38	69.1	3	5.5	McNemar X^2^ = 8.450, *p* = 0.003 *
Adequate compliance	17	30.9	52	94.5	
Mean ± SD	12.6 ± 3.9		21.7 ± 4.1		T = 17.428, *p* < 0.001 **
Effect size (Cohen’s d)	2.821				
95% CI	[−7.031, −5.369]				

T: Paired T-test. * indicates statistical significance at the 5% level (*p* < 0.05); ** indicates statistical significance at the 1% level (*p* < 0.01).

**Table 7:** Correlation among ADHD symptoms, total perceived knowledge, total attitude, and total compliance post-BCW-based nursing intervention implementation.

Items	ADHD Symptoms		Mothers’ Perceived Knowledge		Mothers’ Total Attitude		Mothers’ Total Compliance	
	r	*p*	r	*p*	r	*p*	r	*p*
ADHD Symptoms			−0.399	0.003	−0.434	<0.001 **	−0.328	0.015 *
Mothers’ Perceived Knowledge	−0.399	0.003 *			0.491	<0.001 **	0.451	<0.001 **
Mothers’ Total Attitude	−0.434	<0.001 **	0.491	<0.001 **			0.306	0.023 *
Mothers’ Total Compliance	−0.328	0.015 *	0.451	<0.001 **	0.306	0.023 *		

* indicates statistical significance at the 5% level (*p* < 0.05); ** indicates statistical significance at the 1% level (*p* < 0.01).

## Data Availability

The data presented in this study are available from the corresponding authors upon reasonable request due to privacy and ethical restrictions.
